# Artificial enamel induced by phase transformation of amorphous nanoparticles

**DOI:** 10.1038/s41598-017-02949-w

**Published:** 2017-06-02

**Authors:** Kazuo Onuma, Mayumi Iijima

**Affiliations:** National Institute of Advanced Industrial Science & Technology Central 6, 1-1-1 Higashi, Tsukuba, Ibaraki 305-8566 Japan

## Abstract

Human tooth enamel has tightly packed *c*-axis-oriented hydroxyapatite (HAP: Ca_10_(PO_4_)_6_(OH)_2_) nanorods with high elastic modulus. Fabrication of an enamel architecture *in vitro* supports the repair of teeth using HAP; however, existing methods require complex and laborious steps to form an enamel-like structure. Here we present a very simple and effective technique for forming artificial enamel in near-physiological solution using a substrate composed of amorphous calcium phosphate (ACP) nanoparticles. Without any functionalized modification of the substrate surface, faint dissolution and successive phase transformation automatically induce formation of an intermediate layer of low-crystalline HAP nanoparticles, on which highly oriented HAP nanorods grow by geometrical selection. We also show that an enamel structure forms on a substrate of amorphous calcium carbonate when the surface nanoparticles react so as to form an intermediate layer similar to that in ACP. Our results demonstrate that there is a wide range of substrate choices for nanorod array formation. Contrary to current understanding, a stable surface designed in nanoscale is not essential for the growth of arranged guest crystals. Reactive amorphous nanoparticles and their transformation efficiently induce a nanorod array structure.

## Introduction

Oriented nanocrystals exhibit characteristic physical and chemical properties, as exemplified by the superior potentials of nanorod arrays in metal oxides (ZnO, TiO_2_ and Fe_2_O_3_) as optoelectronic, sensing, energy conversion, and water splitting devices^1–6^. Living beings naturally gained this functional structure in the course of evolution, and oriented nanocrystals developed as hard tissue. For example, the nacreous layer of seashells consists of multiple layers of (001)-oriented aragonite nanoplates (~500 nm thick) bonded by proteins and has beautiful pearlescence^7^. Human tooth enamel consists of *c*-axis-oriented hydroxyapatite (HAP) nanorods (~60 nm wide)^8^, and their hierarchical bundle structure shows high elastic modulus (~60–110 GPa) and hardness (~2–6 GPa)^9, 10^. Fabrication of an enamel structure has been a high-priority target for more than 20 years, and a huge number of trials have been performed^11^. One reason for this is the promise of applying a protocol for artificial enamel fabrication to tooth repair^12, 13^.

It is well known that rod-shaped nano-HAP crystals grow in a calcium phosphate solution containing a small amount of fluoride^14, 15^. The challenge is to construct a nanorod array of oriented HAP on a substrate. Previous efforts to meet this challenge have used either a homogeneous substrate or materials that likely induce HAP as a heterogeneous substrate. These two approaches require complex conditions, such as an extremely low pH^16^, a high temperature^17–23^, the doping of particular organic substances such as enamel protein and a water-soluble matrix extracted from nacre^24–28^, or a functionalized substrate that likely induces HAP^18–20, 23, 29–31^, to obtain an enamel-like structure. Moreover, the grown HAP layers are thin and sometimes poorly oriented. It is evident that an easy-to-use and more effective approach to fabricating a thick enamel-like layer within a short time is strongly required.

Motivated by this situation, we investigated the formation of an artificial enamel layer using ordinary materials in near-physiological calcium phosphate solution without any polymers. We prepared a compression-molded substrate consisting of ACP nanoparticles and utilized its phase transformation to form crystalline HAP nanoparticles^32, 33^ in the solution. We assumed that these crystalline nanoparticles cause geometrical selection^1, 34^ for one-directional growth of HAP rods.

## Results

### Preparation of ACP substrate

A compression-molded calcium phosphate substrate was characterized using field-emission scanning electron microscopy (FE-SEM), micro-beam X-ray diffraction (XRD), and transmission electron microscopy (TEM). The micro-beam XRD pattern of the substrate had a broad low-intensity peak centerd at a 2θ of ~30°, the same as the XRD pattern of calcium phosphate precipitates before compression-molding, which is characteristic of an amorphous material (Supplementary Fig. S1a). An FE-SEM image of a cross-section perpendicular to the substrate surface showed nanoparticles less than 80 nm in size on the inside, estimated by taking into account the thickness of the coated platinum film (~15 nm) (Supplementary Fig. S1b). TEM observations of nanoparticles using a crushed substrate showed no sign of lattice fringes, and the selected-area electron diffraction (SAED) pattern was a faint broad ring (Supplementary Fig. S1c,d), which is consistent with the amorphous nature of particles. The elements in the particles except O were Ca, P, and Cu. The average Ca/P atomic % ratio was 1.33 ± 0.02, measured using scanning-TEM energy-dispersive spectroscopy (STEM-EDS) (Supplementary Fig. S1e). The Cu was attributed to the TEM grid. These findings indicate that the substrate was ACP without any crystalline calcium phosphate phases.

### Fabrication of highly crystalline HAP nanorod array on ACP substrate

**Figure 1:**
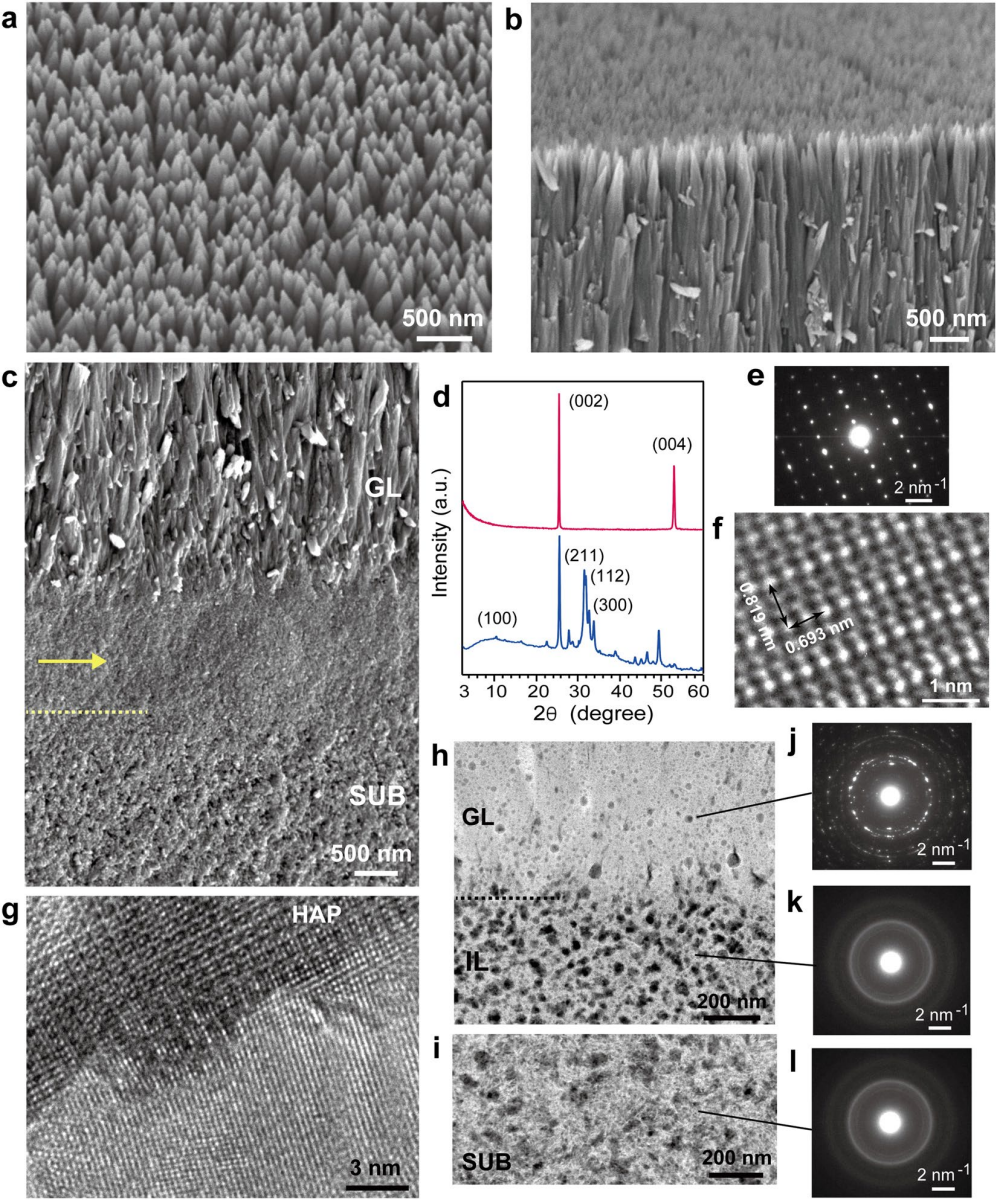
Characterization of ACP substrate immersed for 20 h in mother solution. (**a**) Bird-view SEM image of GL. Cross-sectional SEM images around (**b**) top and (**c**) bottom of GL (arrow: IL; SUB: substrate; dotted line: boundary between IL and SUB). (**d**) XRD (magenta) and micro-beam XRD (blue) patterns of GL. (**e**) SAED pattern of grown crystals, and (**f**) corresponding lattice image. (**g**) Lattice images of HAP rod and portion of transition region. STEM-HAADF images of (**h**) GL and IL and (**i**) inhomogeneous lower substrate. SAED patterns of (**j**) GL, (**k**) IL, and (**l**) inhomogeneous lower substrate.

A thick transparent layer (~20 μm, Supplementary Fig. S1f) grew on an ACP substrate immersed for 20 h in a mother solution containing 0.526 mM F^−^. A bird-view SEM image of the grown layer (GL) showed a tightly packed acute nanorod array (<300 nm wide) (Fig. 1a). The GL contained μm-length high-aspect-ratio (length/width > 10) pillars, arranged in one direction from the initial to final stages of growth (Fig. 1b,c). In the substrate, there was a ~1.2-μm-thick dense intermediate layer (IL) (arrow in Fig. 1c) immediately beneath the GL. The contrast of IL was darker than that of other substrate region, and it was integrated with the rest of the substrate.

A cross-sectional thin film of a grown sample was prepared by ultra-microtome cutting. TEM observation with SAED measurement around the IL revealed that it was not amorphous but randomly oriented crystalline material (Supplementary Fig. S2a–d). SAED measurement also revealed a transition region (~300 nm) between the IL and rod crystals, where the crystallographic orientation gradually changed from random to oriented. The boundary between the IL and transition region was seamless, and the transition region smoothly changed to oriented nanorods.

The XRD pattern of the grown sample had sharp intense peaks at 2θ = 25.9 and 53.3° along with other less remarkable peaks (Fig. 1d, magenta curve). Micro-beam XRD measurement focused on the GL perpendicular to the elongation direction of each rod showed a peak at 2θ = 10.8°, which is characteristic of HAP, and three independent peaks around 2θ = 31–33° (Fig. 1d, blue curve). These findings indicate that the GL consisted of highly crystalline *c*-axis-oriented HAP nanorods that were essentially the same as those in enamel. Since the XRD data showed only average information, the nanoscale structure of each crystal was directly examined by high-resolution TEM (HR-TEM) observation with SAED measurement using a crushed GL. The rod crystals had an ordered atomic arrangement with interplanar distances of 0.819 and 0.693 nm, which correspond to those of HAP (100) and (001), respectively (Fig. 1e,f). The rod HAP was fused with the particles of the transition region, as shown in nanoscale in Fig. 1g, as expected from the low-magnification TEM image in Supplementary Fig. S2. The formation of the HAP nanorod array was independent of the degree of the roughness of initial substrate surface caused by the different compression pressures (5–60 MPa) during substrate preparation (Supplementary Fig. S3).

Since the ultra-microtome cutting frequently destroyed the grown sample, cross-sectional thin film samples were prepared by focused-ion-beam (FIB) treatment. They were used in TEM observation with continuous SAED measurement at steps of ~500 nm from the grown crystal to the substrate. STEM high-angle annular dark field (STEM-HAADF) images showed that the IL had homogeneous contrast despite many voids (back spots in HAADF image) (Fig. 1h). The remainder of the substrate was classified as the upper substrate (US) (<2.5 μm from IL, homogeneous contrast) and the lower substrate (LS) (inhomogeneous contrast due to fibrous pattern) regions (Fig. 1i). In contrast to the SAED pattern of the GL (Fig. 1j), the pattern of the IL was the same as that of the remainder of the substrate, which showed a randomly oriented low-crystalline structure (Fig. 1k,l).

The micro-beam XRD pattern of the substrate was essentially the same as that of the GL. It had a peak at 2θ = 25.8° corresponding to HAP (002) and a small characteristic peak at 2θ = 10.8° corresponding to HAP (100). However, the peak at 2θ = 32° was wide and not separated in contrast to that of the GL (Supplementary Fig. S4a). This indicates that the substrate was low-crystalline HAP, meaning that the IL was also low-crystalline HAP, as indicated by the consistent SAED patterns.

HR-TEM observation showed 10–20-nm crystalline nanoparticles in the IL. A fast Fourier transform image revealed an inner low-intensity ring at 2.90 nm^−1^ from the center and an outer high-intensity ring at 3.57 nm^−1^, consistent with those in the IL SAED pattern (Supplementary Fig. S4b,c). This indicates that 10–20-nm nanoparticles composed the IL. In the remainder of the substrate, fiber crystals (<20 nm wide) were observed with an increase in the depth from the IL. Particularly in the inhomogeneous contrast (LS) region, there were many fibers (Supplementary Fig. S4d,e). The substrate evidently transformed into HAP from ACP due to solution penetration through the pores. The question here is, did the IL also transform from ACP, or did it directly precipitate on the substrate from solution? We performed STEM-EDS analysis, time-resolved SEM observation, and thin-film XRD measurement to answer this question.

### STEM-EDS analysis of grown HAP layer

**Figure 2:**
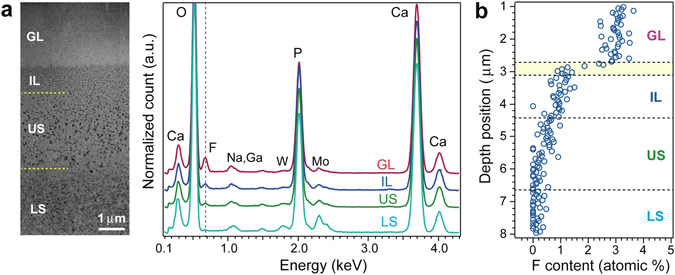
STEM-EDS analysis of ACP substrate immersed for 20 h in mother solution. (**a**) Left: STEM-HAADF image of FIB-prepared cross-section of grown sample (GL: grown layer; IL: intermediate layer; US: upper substrate (homogeneous region); LS: lower substrate (inhomogeneous region)). Dotted lines show boundaries. Right: spectra corresponding to GL, IL, US, and LS. Mo, W, Na, and Ga are attributed to mesh material of TEM grid, supported film used in preparation of FIB-treated sample, sodium acetate buffer in solution, and source of ion beam, respectively. (**b**) One-dimensional F content against depth position. Yellow belt shows transition region.

STEM-EDS analysis showed that the GL and IL contained F^−^ while the remainder of the substrate, especially the inhomogeneous contrast (LS) region, contained very little (Fig. 2a). The average Ca/P atomic % ratios were 1.64 ± 0.02, 1.46 ± 0.02, and 1.50 ± 0.03 for the GL, IL, and the remainder of the substrate (both US and LS regions), respectively. The ratio for the IL was close to that for the substrate. The average fluoride contents were 2.87 ± 0.28, 0.76 ± 0.14, 0.46 ± 0.12, and 0.13 ± 0.09 atomic % for the GL, IL, US region, and LS region, respectively. One-dimensional STEM-EDS analysis from the GL to the LS showed that fluoride content discontinuously decreased in the transition region (shown by yellow belt in figure) and gradually decreased from the IL to the US (Fig. 2b). These data suggest that nanoparticles in the IL did not directly precipitate from the solution.

### Time-resolved SEM observation and thin-film XRD measurement of nanorod array formation process

Time-resolved SEM observations and thin-film XRD measurements were performed to obtain information about the initial stage of growth, during which the IL appeared. A top-view SEM image showed the evidence of particles 50–80 nm in size on the surface due to slight dissolution of the substrate at 1 min after immersion in solution (Supplementary Fig. S5a). The surface changed to a root of tree morphology at 3 min (Supplementary Fig. S5b), and then each root coalesced and a particle-like morphology appeared at 5 min (Supplementary Fig. S5c). From 10 to 20 min, the surface was covered by nanoparticles less than 50 nm in size (Supplementary Fig. S5d,e). The surface morphology changed to nanorods at 30 min, and these rods lengthened and thickened from 30 min to 1 h (Supplementary Fig. S5f,g). A cross-sectional SEM image clearly showed the IL at 5 min (Supplementary Fig. S5h). Elongation of the nanoparticles perpendicular to the substrate started at 10 min; the elongation became enhanced and a column morphology was clearly evident at 20 min (Supplementary Fig. S5i,j). The columns had changed into acute nanorods ~60 nm in width and ~500 nm in length at 30 min (Supplementary Fig. S5k), and the rods were ~120 nm wide and ~900 nm long at 1 h (Supplementary Fig. S5l). Thin-film XRD measurements revealed that the substrate surface gradually changed from ACP to low-crystalline HAP during the first 5 min (Supplementary Fig. S5m). The peak around 2θχ ~ 30° had shifted from the amorphous region to the crystalline one at 3 min, meaning that the appearance of the root of tree structure corresponded to the start of the formation of 10–20-nm low-crystalline HAP nanoparticles in the IL.

Given the elements analysis data, we conclude that the crystalline IL formed via phase transformation from ACP nanoparticles. Thin-film XRD patterns showed a gradual increase in the crystallinity of the grown HAP nanorods from 30 min to 1 h, and a fundamental structure similar to enamel was formed within 1 h.

### Effect of initial Ca/P molar ratio of mother solution on HAP nanorod array structure

Since the initial Ca/P molar ratio of the mother solution (Ca/P = 1.01) might affect the final structure of the HAP nanorod array, we characterized the GL after 20-h immersion of ACP substrates in a modified mother solution with Ca/P = 1.67 or 0.60 while keeping [Ca^2+^] × [(PO_4_)^3−^] equal to 15 (mM)^2^ (Supplementary Fig. S6). The structures of the final HAP nanorod arrays were essentially the same as those formed with Ca/P = 1.01.

### Elastic modulus and hardness of grown HAP layer

The thickness of a grown HAP layer reached ~40 μm while the *c*-axis orientation arrangement remained unchanged during two-day immersion (data not shown). Mechanical strength measurements using nano-indentation equipment showed that the elastic modulus and hardness of this GL, measured perpendicular to the *c*-axis of the crystals, were 63.4 ± 9.2 GPa and 2.87 ± 0.41 GPa (Supplementary Fig. S7). The modulus was smaller, 25–35 GPa, when the indentation direction was parallel to the *c*-axis. These tendencies relative to the indentation direction are opposite those of human enamel^35^. This is attributed to the absence of a bundle structure of rod HAP crystals, which is characteristic of natural enamel, in the present nanorod arrays and to the acute morphology of the HAP that diverted the indentation force. The hardness was also smaller that that of natural enamel (~4 GPa), which was measured using the nanoindenter, compared to that obtained for the indentation direction perpendicular to the *c*-axis^35^.

### Layer grown on ACP substrate in solution without fluoride

**Figure 3:**
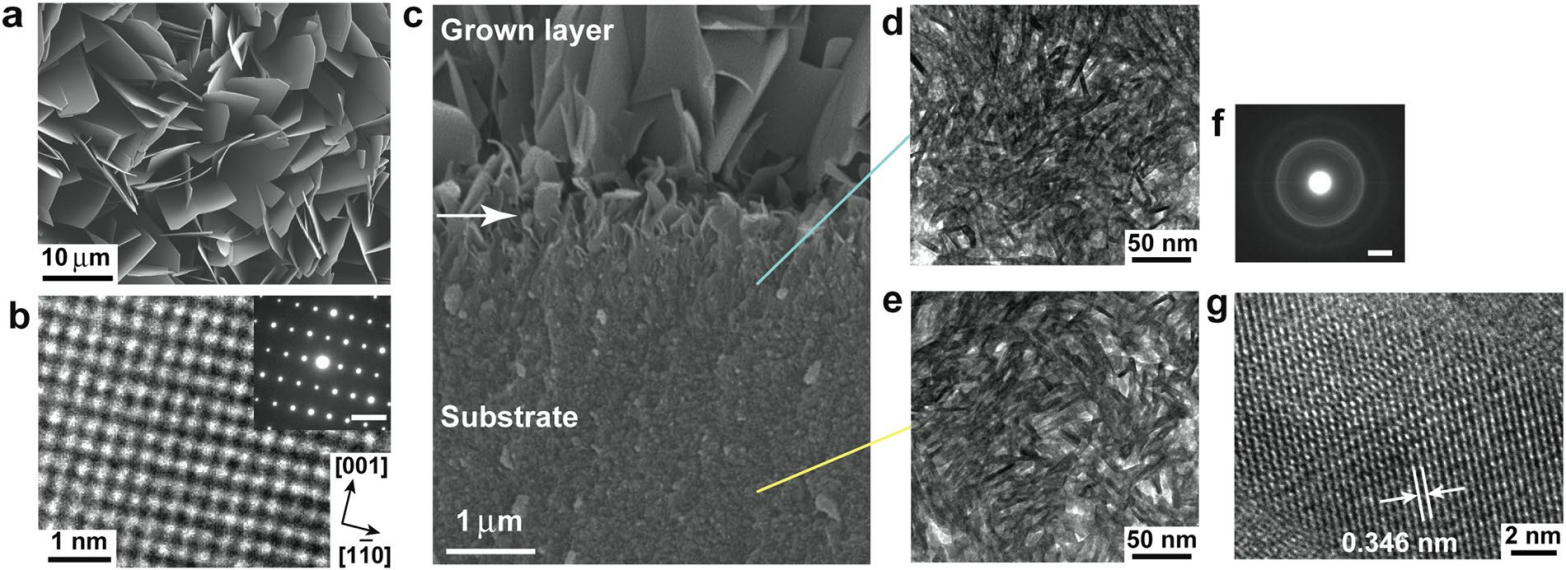
Characterization of ACP substrate immersed for 20 h in fluoride-free mother solution. (**a**) Top-view SEM surface image. (**b**) SAED pattern of grown crystals (upper right, viewed from <110>; scale bar: 2 nm^−1^) and corresponding lattice image. (**c**) Cross-sectional SEM image (arrow: fragmented crystals). TEM images: (**d**) beneath fragmented crystals and (**e**) in deep region of substrate. Fiber crystals were observed everywhere; IL was not observed. (**f**) SAED pattern of substrate consistent with that in Fig. 1l (randomly oriented low-crystalline HAP). Scale bar: 2 nm^−1^. (**g**) HR-TEM image of fiber in substrate. Fringe spacing corresponds to HAP (002).

We investigated how fluoride in the solution was related to the formation of the IL and subsequent enamel structure. After 20-h immersion of an ACP substrate in a mother solution without fluoride, the surface morphology had drastically changed, and an aggregation of thin plates 10–15 μm wide had developed on the surface, as seen in a top-view SEM image (Fig. 3a). XRD and micro-beam XRD measurements showed that the GL consisted of *c*-axis-oriented highly crystalline octacalcium phosphate (Ca_8_(HPO_4_)_2_(PO_4_)·5H_2_O; OCP) (Supplementary Fig. S8a, magenta and blue curves). The orientation degree was less than that of a HAP nanorod array, as seen in the appearance of peaks around 2θ ~ 32°, except for the intense (002) and (004) peaks in the XRD pattern (magenta curve in Supplementary Fig. S8a). The SAED pattern and an HR-TEM image of the grown crystals from a crushed sample showed diffraction spots and lattice fringes corresponding to interplanar distances of 0.949 and 0.690 nm (Fig. 3b). These values correspond to the OCP *b*- and *c*-axes respectively although the *b*-axis was elongated (~5%), probably due to the incorporation of acetic acid molecules in the H_2_O layer of the OCP structure.

To clarify why HAP did not grow, we characterized the substrate in detail. Micro-beam XRD measurement of a substrate sample after 20-h immersion showed that major portions of the substrate had transformed into low-crystalline HAP and that a small amount of OCP coexisted with HAP, as seen in the weak shoulder peak at 2θ = 4.7°, characteristic of OCP (ocher curve in Supplementary Fig. S8a). This is in stark contrast to the OCP growth on the substrate. Since the peak at 2θ = 4.7° in the pattern was not observed for substrates immersed for 30 min or 1 h (data not shown), transformation from ACP to HAP via OCP in the initial growth stage did not occur. The OCP in the substrate formed later than the HAP. An SEM image of a cross-section of a grown sample showed that the contrast of the substrate was homogeneous from deep region to the surface and that OCP crystals, including initial fragmented crystals (arrow), grew directly on the substrate (Fig. 3c). The region where the fragmented crystals were observed corresponds to the transition region in the HAP growth (see Supplementary Fig. S2); however, there was no IL beneath these crystals. This suggests that the presence or absence of an IL controls HAP growth on a substrate. We therefore performed time-resolved SEM observations and XRD measurements.

At 1 min after substrate immersion, many fragmented precipitates appeared on the surface (Supplementary Fig. S8b). ACP nanoparticles of the original surface became visible, meaning that the surface slowly dissolved. The number of fragmented precipitates increased and covered the surface at 3 min (Supplementary Fig. S8c). A structure similar to the root of tree one observed in the HAP growth appeared (Supplementary Fig. S8c,d); however, it did not change into nanoparticles, and subsequent elongation perpendicular to the substrate did not occur due to the growth of fragmented precipitates. These precipitates coalesced over time and had formed plates at 10 min although the plate edges were not sharp. The plate morphology was the same as that of the final OCP at 1 h (Supplementary Fig. S8e–g).

Time-resolved thin-film XRD measurements of the substrate surface showed that it gradually changed from ACP to OCP without the appearance of HAP (data not shown). We conclude that the initial fragmented precipitates on the surface were low-crystalline OCP. Their rapid precipitation, growth, and transformation into highly crystalline OCP hindered formation of HAP nanoparticles in the IL and their elongation to the substrate.

### HAP nanorod array on amorphous calcium carbonate substrate

**Figure 4:**
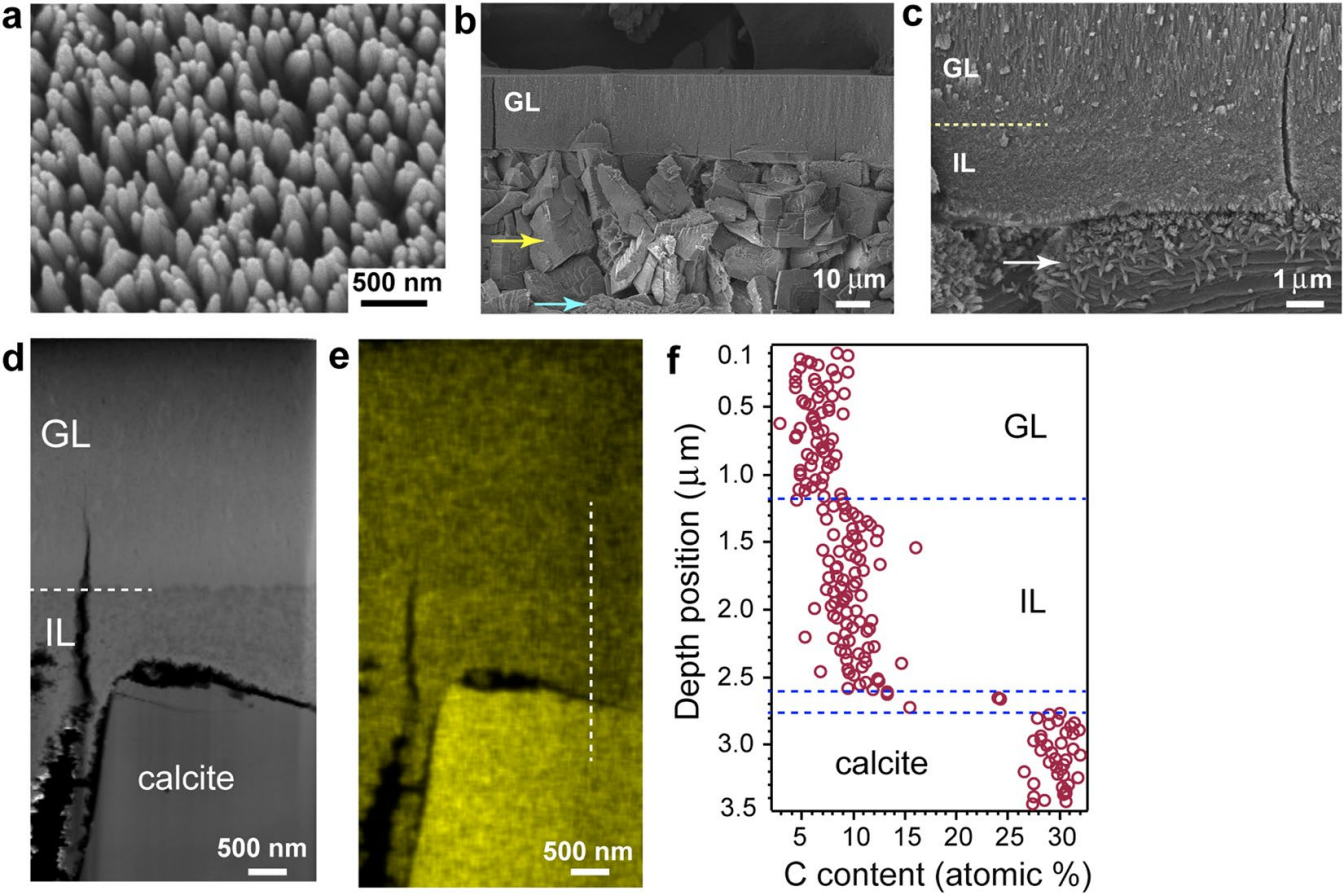
SEM observation and STEM-EDS analysis of ACC substrate immersed for 20 h in mother solution. (**a**) Bird-view SEM image of grown surface. (**b**) Cross-sectional image of sample (GL: grown layer; yellow arrow: calcite; blue arrow: vaterite). (**c**) Magnified image of (**b**). Dotted line: boundary between GL and IL; arrow: HAP (Supplementary Fig. S10). (**d**) STEM-HAADF image of FIB-prepared cross-sectional thin film. (**e**) STEM-EDS elemental mapping of C in same area as HAADF image. (**f**) One-dimensional C content against depth position measured along dotted line in (**e**).

To clarify whether formation of the IL is characteristic of an ACP substrate, we prepared a heterogeneous substrate consisting of amorphous calcium carbonate (ACC; CaCO_3_) nanoparticles (Supplementary Fig. S9) and immersed it in the same mother solution (containing 0.526 mM fluoride) used for the ACP substrate. Figure 4a shows a top-view SEM image of the ACC substrate after 20-h immersion. A nanorod array similar to those observed on the ACP substrate had formed. The thickness of the GL was ~25 μm, as estimated from a cross-sectional SEM image (Fig. 4b). The GL was highly crystalline HAP oriented to the *c*-axis, as revealed by XRD and micro-beam XRD measurements (Supplementary Fig. S10a).

The ACC substrate had transformed into crystalline phases during growth. Cubic (yellow arrow in Fig. 4b) and spherulitic (blue arrow) materials were observed in the substrate. The micro-beam XRD pattern of the substrate had peaks corresponding to both calcite and vaterite (Supplementary Fig. S10b). A massive layer was observed immediately beneath the HAP layer, as shown by the magnified image of the cross-section (Fig. 4c). HR-TEM observation and SAED measurement using an FIB-treated sample revealed that this layer had the same characteristics observed for the IL in the ACP substrate. It consisted of randomly oriented low-crystalline HAP nanoparticles 10–20 nm in size (Supplementary Fig. S10c,d). HR-TEM observation and the SAED pattern for the cubic materials in the substrate showed that they were calcite crystals (Supplementary Fig. S10e,f). The lattice image observed on the calcite corresponded to that of HAP (Supplementary Fig. S10g), indicating that the low-aspect nanorods observed on the cubic crystal (arrow in Fig. 4c) were HAP.

Time-resolved SEM observations of nanorod array formation showed that the ACC nanoparticles on the surface dissolved during the initial stage (less than 10 min, Supplementary Fig. S11a–c) and that a root of tree structure had formed at 10 min (Supplementary Fig. S11d). The roots gradually changed into nanoparticles without random precipitation of materials from the solution (Supplementary Fig. S11e). These nanoparticles grew and elongated perpendicular to the substrate over time, which led to acute nanorods at 1 h (Supplementary Fig. S11f). This nanorod formation process is essentially the same as that observed for the ACP substrate. Time-resolved SEM observation suggested that the dissolving ACC nanoparticles released calcium and carbonate ions near the surface that increased the supersaturation with respect to HAP, which caused overgrowth of HAP around the ACC nanoparticles, resulting in the formation of an IL.

STEM-EDS analysis from the GL to the substrate using an FIB-treated sample showed that the average Ca/P atomic % ratios of the GL and IL differed from those using the ACP substrate (Supplementary Fig. S11g). They were 1.69 ± 0.02 for the GL and 1.96 ± 0.03 for the IL. Elemental mapping and the one-dimensional profile of carbon showed higher content in the IL than in the GL (Fig. 4d–f), consistent with the assumption that HAP nanoparticles in the IL overgrew on ACC nanoparticles. The fluoride content in the GL was comparable to that using an ACP substrate, 2.75 ± 0.27%; however, that in the IL was 1.36 ± 0.17%, 1.8 times that using an ACP (Supplementary Fig. S11g). This also supports the assumption that the IL formed via overgrowth of HAP on original ACC nanoparticles, which differs from the direct transformation from ACP nanoparticles into HAP ones observed for the IL using an ACP substrate. When we used a cleaved calcite (10–14) surface or compression-molded calcite microcrystals as the substrate, highly oriented HAP nanorod arrays did not form on the substrate (data not shown), indicating the importance of amorphous nanoparticles in the fabrication of a HAP nanorod array structure.

## Discussion

The reaction of an ACP substrate in solution with and without fluoride revealed two factors contributing to the formation of an enamel architecture: the level of F^−^ in the solution and an IL consisting of densely packed low-crystalline HAP nanoparticles. These two factors are related. The dense packing and the crystalline nanoparticle nature of the IL likely caused free growth of surface particles perpendicular to the substrate by geometrical selection, as evidenced by the presence of a transition region. Gradually increasing fluoride incorporation into the particles during growth contributed to the high crystallinity of the HAP nanoparticles, which accelerated the growth toward the *c*-axis and resulted in oriented HAP nanorods (see Supplementary Fig. S5). As seen in the results of the time-resolved SEM observations and thin-film XRD measurements made during the initial reaction stages (Supplementary Fig. S5), there was no sign of direct precipitation of HAP nanoparticles in the IL formation. The data show that ACP nanoparticles slightly dissolved and directly transformed into low-crystalline HAP nanoparticles, forming the IL. This is consistent with our previous light scattering investigations that showed direct structural phase transformation between ACP and HAP^32, 33^. The presence of fluoride in the solution caused formation of (fluoride-substituted) HAP as the thermodynamically stable phase, although the weakly acidic pH of the solution is favourable for OCP formation, as seen in the data obtained under the fluoride-less condition (Fig. 3). This is due to the much lower solubility of fluoride-substituted HAP than that of OCP. The solubilities (−log (K_sp_)) at 37 °C of OCP, HAP, and FAP (fluorapatite) are 98.6, 117.2, and 122.3, respectively^36^. OCP is much more soluble than FAP.

An early study in this field showed that the solubility of fluoride-substituted HAP (Ca_10_(PO_4_)_6_(OH)_2−x_(F)_x_) is minimum at x = 1.12^37^. STEM-EDS analysis showed that the x component of the present HAP nanorod arrays was ~0.8. We concluded that the present fluoride-substituted HAP grew as the most stable phase in the mother solution.

After the formation of HAP nanorods began, nucleation and homo-epitaxial growth of HAP on the initial nanorods occurred repeatedly, resulting in a thick enamel-like HAP layer. At this stage, of course, (macroscopic) geometrical selection for each crystal effectively contributed to the growth perpendicular to the substrate.

Use of less-reactive crystalline nanoparticles (HAP, β-tricalcium phosphate, and TiO_2_) of various sizes for the substrate also induced HAP nanorod arrays. However, frequent spherulitic growth reduced the homogeneity and *c*-axis orientation degree of the GL (Supplementary Fig. S12). This indicates that formation of a HAP nanorod array structure is not simply controlled by the size of the nanoparticles although nanoparticles are an essential factor in nanorod array fabrication. We assume that a surface energy gap between the fluoride-free nanoparticles in the substrate and the grown fluoride-containing HAP nanorods inhibited the efficient geometrical selection for an oriented nanorod arrays during the initial growth stage in the case of crystalline nanoparticles. Given the laborious processes for preparing homogeneous crystalline nanoparticles, reactive amorphous nanoparticles are greatly advantageous for fabricating an enamel architecture.

Contrary to the common understanding, our study showed that the surface design of a substrate in nanoscale or use of a highly oriented single crystal surface is not necessary for inducing the growth of a HAP nanorod array. Simple compression-molding of reactive amorphous nanoparticles containing a calcium element effectively induced the formation of an IL composed of densely packed HAP nanoparticles, on which an enamel-like structure immediately formed even under near-physiological conditions. The substrate material is less important than the amorphous nature of the substrate. Since preparation of amorphous nanoparticles is much easier than that of crystalline phases, our technique is widely applicable to fabrication of nanorod arrays in addition to HAP ones.

## Methods

### Calcium phosphate solution for ACP nanoparticle preparation

Reagent grade CaCl_2_ or Na_2_HPO_4_ (Nacalai Tesque) was dissolved in Milli-Q water (18.2 MΩ, TOC: 3 ppb) to obtain solutions with a concentration of 10 mM. Solutions were placed in incubator at 4 °C and then mixed together with stirring for 1 min to 1:1 volume ratio. The mixed solution was vacuum-filtered using 0.22-μm-pore cellulose-acetate filter (Advance) at room temperature (~23 °C). Precipitates on the filter were quickly washed with Milli-Q water to remove residual solution and then with 99.5% ethanol (Wako, analytical grade) to stop further reaction. After being frozen at −70 °C for 30 min, ACP precipitates were vacuum-dried overnight and placed in a dry incubator (~20% humidity at ~23 °C) until use.

### Calcium carbonate solution for ACC nanoparticle preparation

Reagent grade CaCl_2_ or Na_2_CO_3_ (Nacalai Tesque) was dissolved in Mill-Q water (18.2 MΩ, TOC: 3 ppb) to obtain solutions with a concentration of 20 mM. Solutions were mixed together with stirring at 4 °C for 1 min to 1:1 volume ratio. Procedure subsequently used to obtain ACC precipitates was the same as that for ACP. ACC precipitates were stored at −20 °C until use to prevent transformation into calcite or vaterite.

### ACP and ACC substrates

Approximately 10 mg of ACP or ACC nanoparticles were molded to form 2 × 10 × 0.5 mm (thickness) substrate using hydraulic oil compressor at 20 MPa for 10 min. Substrate was then cut into 2 × 2 × 0.5 mm fragments with surgical knife for use. Immediately before each nanoparticle was molded into substrate, powder XRD measurement was done to compare the pattern with that obtained immediately after nanoparticle preparation.

### Crystalline nanoparticle substrates

Approximately 10 mg of commercially available HAP (Taihei Chemical Industrial, average particle size <100 nm), β-tricalcium phosphate, and commercially available TiO_2_ (TECNAN, average particle size ~10–15 nm, anatase/rutile mixture) nanoparticles were compression-molded at 20 MPa for use as substrates. The β-tricalcium phosphate nanoparticles were prepared by atomization (Easy-Nano, AMEX) of commercially available microcrystals (Wako) using ZrO_2_ beads (ϕ = 30 μm) in 99.5% ethanol solution. Resulting slurry was centrifuged for 30 min at 15,000 rpm at 4 °C; the supernatant was filtered and dried to obtain nanoparticles (average size <60 nm).

### Mother solution for substrate immersion

Stock solutions of 1.00 M CaCl_2_, 0.50 M K_2_HPO_4_, 0.50 M KH_2_PO_4_, 1.00 M CH_3_COOH, 0.10 M CH_3_COONa, and 52.6 mM NaF (corresponding to 1,000 ppm F^−^) were prepared by dissolving each reagent in Milli-Q water. After each solution was filtered using 0.22-μm-pore cellulose-acetate filter (Advance Co.), 4.00-mM phosphate solution was made by mixing K_2_HPO_4_ with KH_2_PO_4_ at a molar ratio of 1:1, which was buffered to a pH of 6.30 ± 0.01 (at 25 °C) using stock CH_3_COONa and CH_3_COOH solutions. The concentration of CH_3_COO^−^ in buffered phosphate solution was 50 mM. Aliquot of 1.00 M CaCl_2_ stock solution was then mixed with buffered phosphate solution at a volume ratio of 1:249 to achieve calcium-ion concentration of 4.00 mM. This reduced the phosphate-ion concentration in the mixed solution to 3.98 mM. This calcium phosphate solution was mixed with 52.6 mM stock NaF solution at a volume ratio of 99:1 to obtain mother solution with final F^−^ concentration of 0.526 mM (corresponding to 10 ppm). The calcium- and phosphate-ion concentrations in the final mother solution were 3.96 and 3.94 mM, respectively, and the pH of mother solution was 6.20 ± 0.01. Approximately 4-mM concentrations for the calcium and phosphate ions in the mother solution are close to the upper limit at which the solution could be kept for two days without three-dimensional nucleation (precipitation). Approximately 35 mL of the mother solution was poured into polystyrene vessel (~50 mL volume), which was sealed with lid and placed in incubator (37.0 ± 0.5 °C). The substrate was placed at the vessel bottom, with the substrate surface parallel to the bottom. Volume ratio between the mother solution and substrate was ~17,500:1. The mother solution was not stirred during substrate immersion. After the target immersion time, the substrate was quickly removed and washed with Milli-Q water and then with 99.5% ethanol and dried in air.

### XRD

Powder X-ray diffractometer (RINT 2000, Rigaku, monochromated CuKα) was used at 40 kV and 100 mA to characterize substrates immersed for 20 h in the mother solution or in the same solution without fluoride. Samples were placed in non-reflective quartz sample holder and scanned at 2θ from 3 to 60° with a scanning speed of 2° min^−1^. The peak positions of the measured samples were referenced to those of calcium phosphates or calcium carbonates in JCPDS cards.

### Micro-beam XRD

Cross-sections (~200–400 μm thick) of grown sample cut perpendicular to the substrate surface were prepared and characterized using micro-beam X-ray diffractometer with monochromated CuKα. Beam focused to a 100-μm diameter using collimator (RAPID, Rigaku) was irradiated perpendicular to the section surface of GL or substrate for material characterization at 50 kV and 30 mA. The sample was fixed on top of glass capillary using double-stick tape, and the diffraction was recorded on imaging plate. Digital data on the imaging plate were converted into intensity vs. 2θ relationship using DISPLAY software (Rigaku). Final peak positions of the samples were referenced to those of calcium phosphates or calcium carbonate in JCPDS cards.

### Thin-film in-plane XRD

Grown surface was characterized using thin-film XRD with Cu target at 45 kV and 200 mA (SmartLab, Rigaku, monochromated CuKα). Thin-film XRD measurements were done using 2 × 5 × 0.5 mm (thickness) substrates. The angle of incident beam on the surface was 0.2°, and the sample was scanned at 2θχ from 3 to 50° with a scanning speed of 0.1° min^−1^. The peak positions of the samples were referenced to those of calcium phosphates or calcium carbonates in JCPDS cards.

### FE-SEM

Samples (GL with substrate) were observed after platinum coating using FE-SEM (JSM-7000F, JEOL) with an acceleration voltage of 5 kV. To avoid sample charging, the thickness of coated film was set to ~15 nm.

### Crushed samples for TEM observation

GL or substrate was crushed in agate mortar and mixed with 99.5% ethanol. For observation of the GL, it was separated from the substrate by cutting at GL-substrate interface using surgical knife under optical microscope. The crushed sample was dispersed in ethanol solution by ultrasonic bathing for 5 min at 40 kHz. Aliquot was placed on TEM grid with Cu mesh and allowed to dry naturally in air.

### FIB-prepared samples for TEM observation

Cross-sectional thin film was prepared using conventional FIB technique with Ga ion source (FB-2100, Hitachi). The samples were thinned to ~150 nm at acceleration voltage of 40 kV and then to ~100 nm at 10 kV. Prepared samples were placed on TEM grid with Mo mesh for observation.

### Ultra-microtone-prepared samples for TEM observation

Samples were embedded in epoxy resin and placed in incubator for 72 h at 50 °C to solidify them. The solidified blocks were sliced with diamond knife to a thickness of 70–100 nm. The sliced samples were placed on TEM grid with Cu mesh for observation.

### TEM

Analytical TEM (Tecnai Osiris, FEI) was used for sample observations at an acceleration voltage of 200 kV. SAED measurements were performed for selected area: 200 nm ϕ for the transition region and 800 nm ϕ otherwise (Supplementary Fig. S2).

### STEM-EDS

High-speed two-dimensional elemental analysis was performed using Super-X EDS system in the TEM. Four X-ray detectors (silicon drift detectors) were arranged close to the sample for rapid and accurate measurement. The probe diameter of electron beam used for elemental mapping in STEM mode was ~0.5 nm, and the probe amplitude was ~0.55 nA. The beam residence time at each measured position was 10 μsec, and mapping was completed within 5 min. Mapping data were converted into energy-dependent spectra by using peak deconvolution method. Error appearing in the final content of each element was attributed to calculation error in peak deconvolution.

### STEM-HAADF

Pre-specimen lenses in TEM focused the electron beam into a small probe (~1 nm diameter) that was scanned in raster pattern across FIB-prepared cross-sectional sample. The images were obtained by collecting transmission electrons scattered to higher angles using annular detector. The contrast in STEM-HAADF image depends on the intensity of scattered electrons in the sample.

### Optical microscopy

Confocal differential-interference-contrast microscopy (OLS4100, OLYMPUS Co. Ltd.) with white light was used for observing cross-sectional-cut ACP substrate after 20-h immersion in mother solution.

### Atomic force microscopy

MulitiMode-8 atomic force microscopy (AFM) (Bruker AXS) was used for observing substrate surface depending on compression pressure before immersion in mother solution (Supplementary Fig. S3). Silicon single-crystal cantilever (spring constant = 0.4 Nm^−1^, tip curvature radius ~2 nm) was used in peak-force tapping mode. Typical scanning condition was 2 Hz and 512 scan lines. Ten different 5 × 5 μm areas around the center of substrate were observed, and the surface roughness was estimated using NanoScope Analysis software (ver. 1.4, Bruker AXS) after correcting for the inclination of raw image. The average roughness (R_q_) and its standard deviation for each substrate were calculated.

### Mechanical strength test

Elastic modulus and hardness of HAP layer on ACP substrate after two days of immersion in mother solution were measured using nanoindenter (Nano Indenter 2000, Agilent Technologies) with a Berkovich probe. The nanoindenter was calibrated using fused silica plate before measurement of the HAP layer. Top curvature radius of the probe was 20 nm, and top angle of the triangular pyramid was 135°. Measurements were done in continuous stiffness mode, and dependence of the elastic modulus and hardness on the indentation depth was calculated by analyzing load vs. displacement curve. The indentation direction was perpendicular to elongation direction of each nanorod in the HAP layer. Fourteen points were measured for three samples, and the average and standard deviation of the elastic modulus and hardness were calculated.

### Data availability

All relevant data are available upon request. Please address requests to Dr Kazuo Onuma.

## Electronic supplementary material

Supplementary information
