# Supplementary material for: Artificial enamel induced by phase transformation of amorphous nanoparticles
Source: Sci Rep. 2017 Jun 2;7:2711. doi: 10.1038/s41598-017-02949-w (PMC5457434; doi:10.1038/s41598-017-02949-w)
Supplement: Supplementary file 1 — Supplementary information [file 41598_2017_2949_MOESM1_ESM.pdf]

Supplementary Information

Artificial enamel induced by phase transformation of amorphous nanoparticles

Kazuo Onuma* and Mayumi Iijima

National Institute of Advanced Industrial Science & Technology, Central 6

1-1-1 Higashi, Tsukuba, Ibaraki 305-8566 Japan

***Corresponding author:**

Kazuo Onuma, PhD

Upper Senior Researcher

National Institute of Advanced Industrial Science & Technology, Central 6

1-1-1 Higashi, Tsukuba

Ibaraki 305-8566, JAPAN

Tel: +81-29-861-4832; Fax: +81-29-573861-6149

E-mail: k.onuma@aist.go.jp

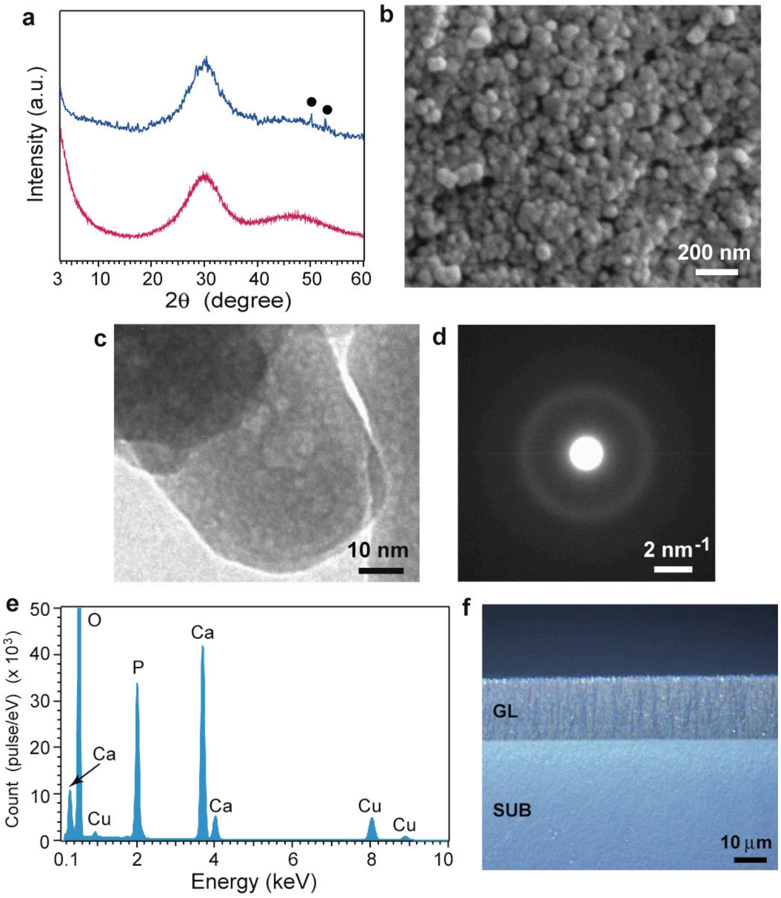

Supplementary Fig. S1. Characterization of ACP substrate before immersion in mother solution and cross-sectional optical microscope image of grown sample after 20-h-immersion. (**a**) XRD pattern of ACP powder before molding (magenta), and micro-beam XRD pattern of cross-section of compression-molded substrate (blue). Spikes indicated by solid circles are from damage in imaging plate, not from sample. (**b**) Cross-sectional SEM image of substrate. Nanoparticle size is less than 80 nm. (**c**) TEM image of nanoparticles in substrate observed using crushed sample. (**d**) SAED pattern of nanoparticles. (**e**) STEM-EDS analysis of

elements in substrate. Average Ca/P atomic % ratio of substrate was 1.33±0.02. Cu is attributed to mesh material of TEM grid. (**f**) Confocal differential-interference-contrast microscope image of cross-section of grown sample (GL: grown layer, SUB: substrate).

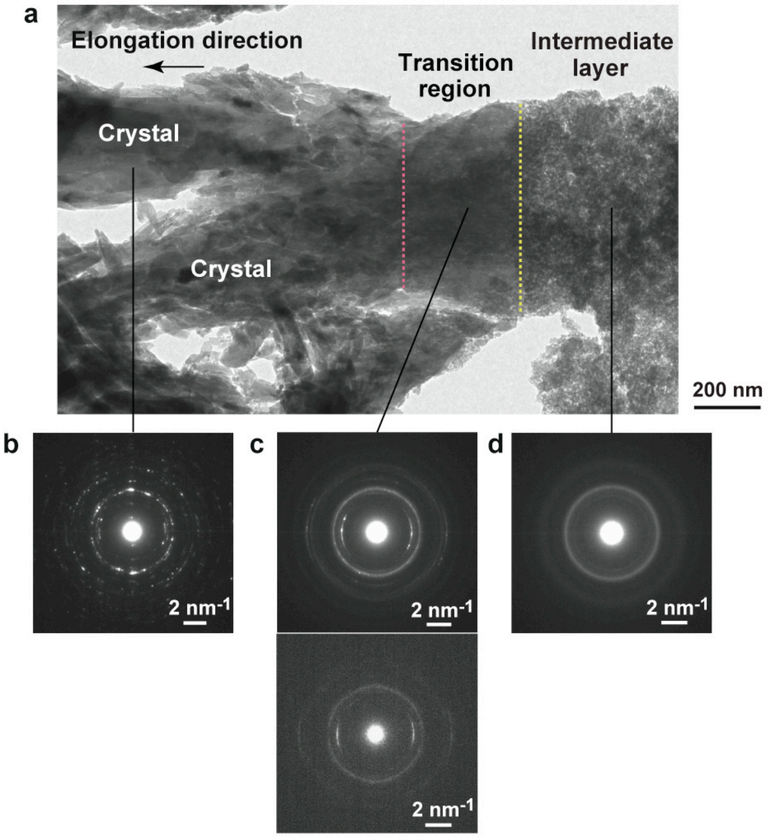

Supplementary Fig. S2. TEM image and SAED pattern of grown sample immersed in mother solution for 20 h prepared using ultra-microtome cutting. (**a**) TEM image of grown crystals, transition region, and intermediate layer. Magenta and yellow dotted lines are boundary between grown crystals and transition region and that between transition region and intermediate layer, respectively. SAED patterns corresponding to (**b**) grown crystals, (**c**) transition region, and (**d**) intermediate layer. Lower pattern for transition region is for 200 nm ϕ area; it is essentially the same as that for 800 nm ϕ area (upper pattern).

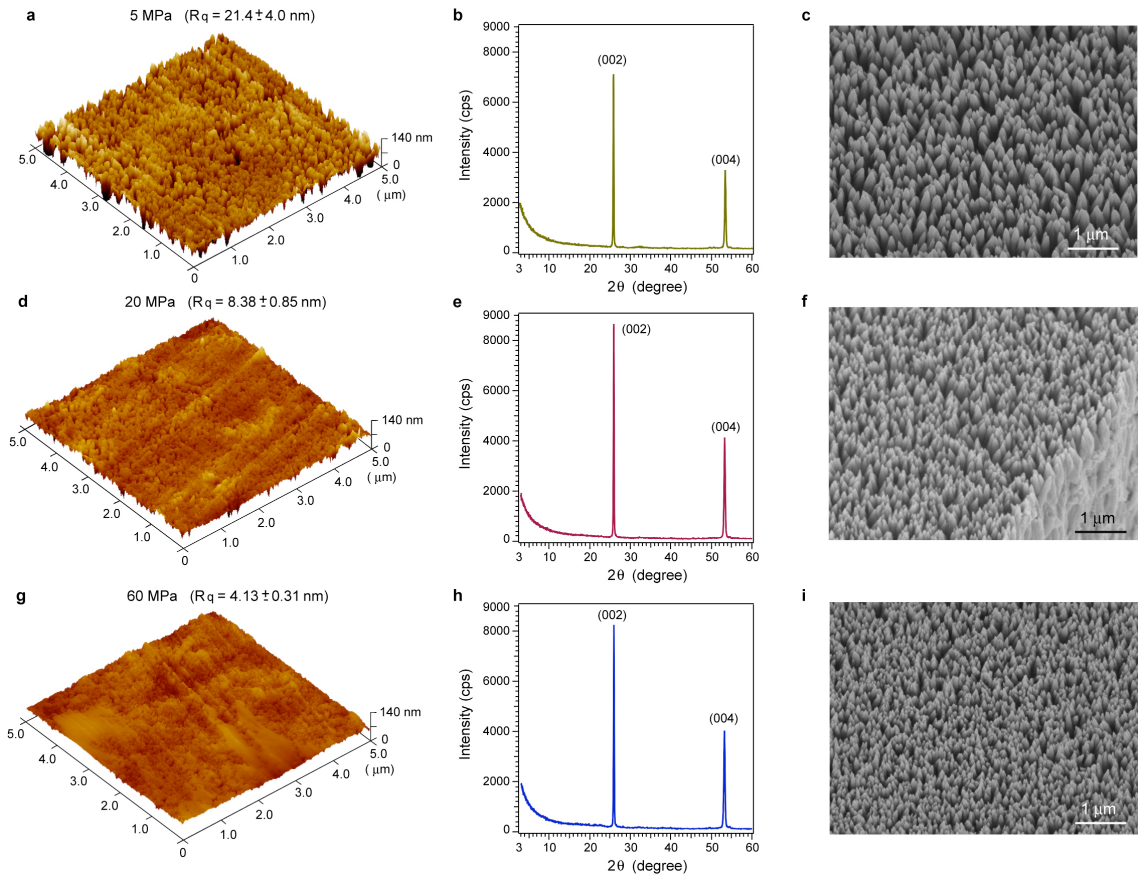

Supplementary Fig. S3. Characterization of initial ACP substrate and GL after 20-h immersion in mother solution for three compression pressures. (**a**) AFM image of substrate surface prepared at 5 MPa with measured average roughness (R_q) and standard deviation. (**b**) XRD pattern of 5-MPa substrate after immersion. (**c**) Bird-view SEM image of GL on 5-MPa substrate after immersion. (**d**) AFM image of substrate surface prepared at 20 MPa. (**e**) XRD pattern of 20-MPa substrate after immersion. (**f**) Bird-view SEM image of GL on 20-MPa substrate after immersion. (**g**) AFM image of substrate surface prepared at 60 MPa. (**h**) XRD pattern of 60-MPa substrate after immersion. (**i**) Bird-view SEM image of GL on 60-MPa

substrate after immersion. Width of each nanorod decreased with increase in compression pressure.

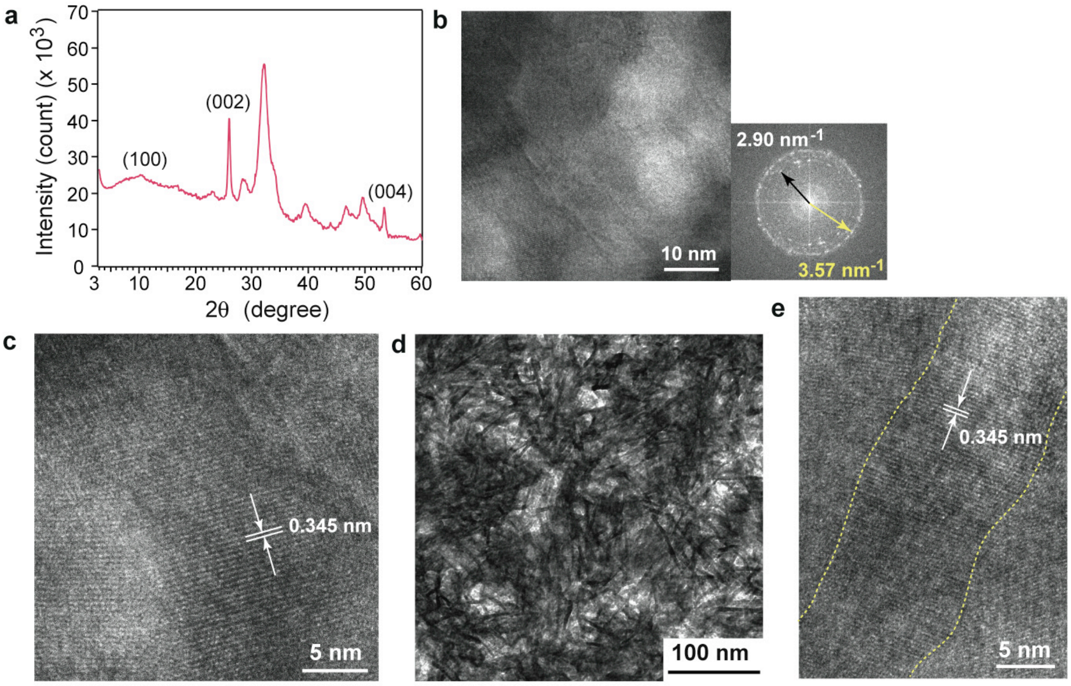

Supplementary Fig. S4. Characterization of substrate and intermediate layer after 20-h immersion of ACP substrate in mother solution. (**a**) Micro-beam XRD pattern of substrate after immersion. All peaks are attributed to HAP. (**b**) TEM image of intermediate layer (left) and corresponding fast Fourier transform image (right). Nanoparticles 10–20 nm in size were observed in intermediate layer. (**c**) HR-TEM image of nanoparticles. Fringe spacing corresponds to HAP (002). (**d**) Fiber crystals in inhomogeneous contrast region of substrate. (**e**) HR-TEM image of fiber. Yellow dotted curves are fiber boundaries.

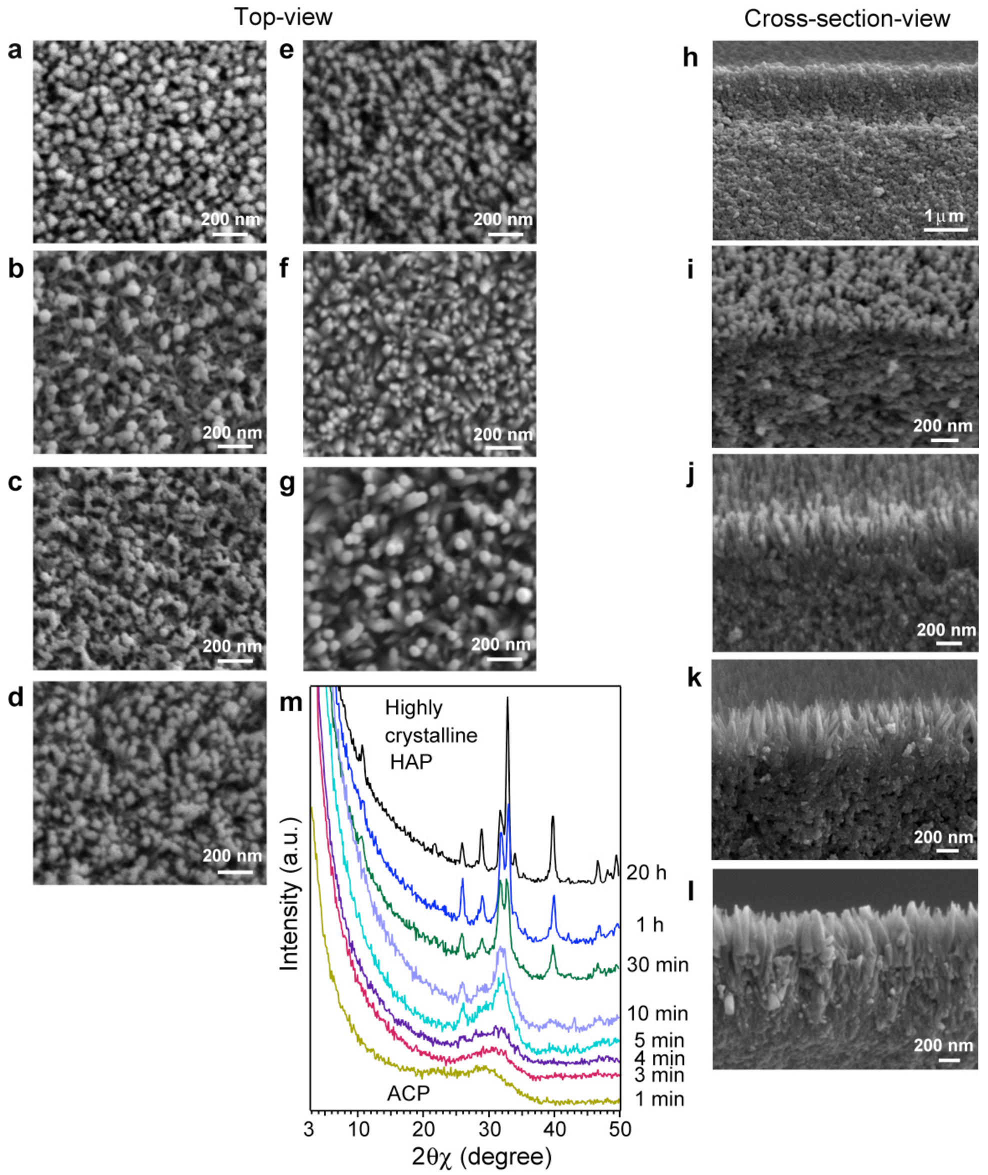

Supplementary Fig. S5. Time-resolved SEM observation and thin-film XRD measurement of ACP substrate immersed in mother solution. Top-view SEM images after (**a**) 1-min, (**b**) 3-min, (**c**) 5-min, (**d**) 10-min, (**e**) 20-min, (**f**) 30-min, and (**g**) 1-h immersion. Surface consisting of root of trees at 3 min changed to one consisting of nanoparticles between 5 and 20 min and then to one consisting of nanorods at 30 min. Cross-section-view SEM images after (**h**) 5-min,

(**i**) 10-min, (**j**) 20-min, (**k**) 30-min, and (**l**) 1-h immersion. (**m**) Thin-film XRD patterns of surface over time.

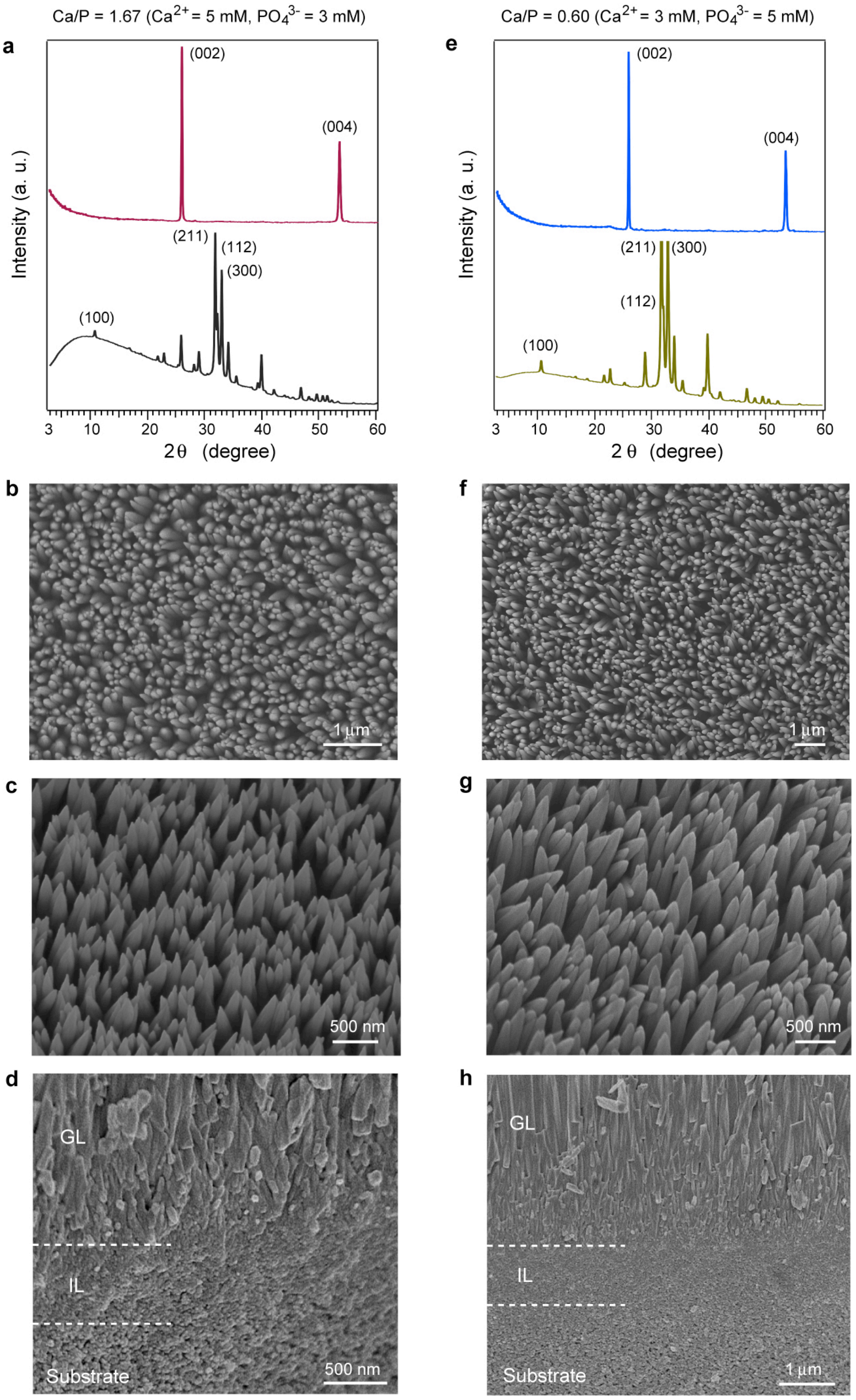

Supplementary Fig. S6. Characterization of GL for two initial Ca/P molar ratios in mother solution. (**a**) XRD (magenta) and micro-beam XRD (black) patterns of GL grown in Ca/P = 1.67 mother solution (Ca^{2+} = 5 mM, PO_4^{3-} = 3 mM). GL was highly crystalline *c*-axis

oriented HAP. (**b**) Top-view SEM image of GL (Ca/P = 1.67). (**c**) Bird-view SEM image of GL in final stage of growth (Ca/P =1.67). (**d**) Cross-sectional SEM image of initial stage of growth (Ca/P = 1.67). Intermediate layer (IL) was seen, and rod HAP crystals arranged in one direction. (**e**) XRD (blue) and micro-beam XRD (ochre) patterns of GL in Ca/P = 0.60 mother solution (Ca^{2+} = 3 mM, PO_4^{3-} = 5 mM). GL was highly crystalline *c*-axis oriented HAP. (**f**) Top-view SEM image of GL (Ca/P = 0.60). (**g**) Bird-view SEM image of GL in final stage of growth (Ca/P =0.60). (**h**) Cross-sectional SEM image of initial stage of growth (Ca/P = 0.60). GL consisted of HAP nanorod array structure independent of initial Ca/P ratio in mother solution

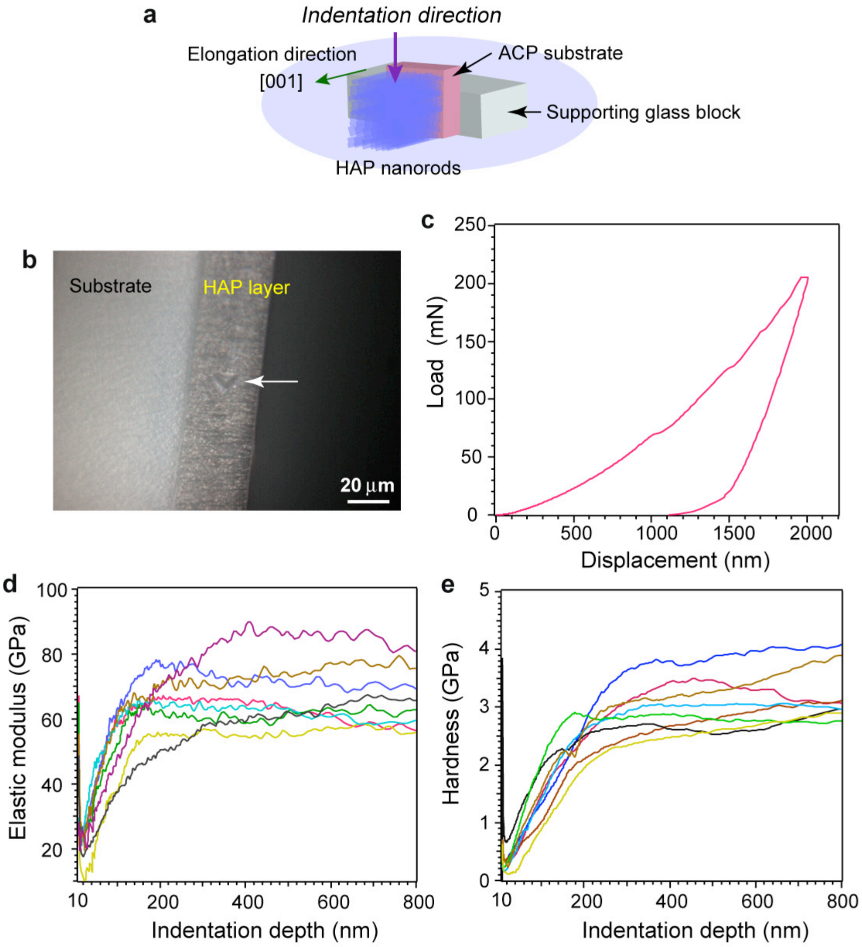

Supplementary Fig. S7. Mechanical strength measurement of HAP layer formed after two days of immersion of ACP substrate in mother solution. (**a**) Illustration of sample and indentation direction. Thickness of HAP layer was ~35–40 µm. (**b**) Photograph of indentation produced (arrow). (**c**) Example load vs. displacement curve. (**d**) Elastic modulus vs. indentation depth profiles (eight representative data curves). (**e**) Hardness vs. indentation depth profiles (eight representative data curves using same samples as in (**d**)). Elastic modulus and hardness were estimated by averaging the data between 400 and 600 nm for each sample.

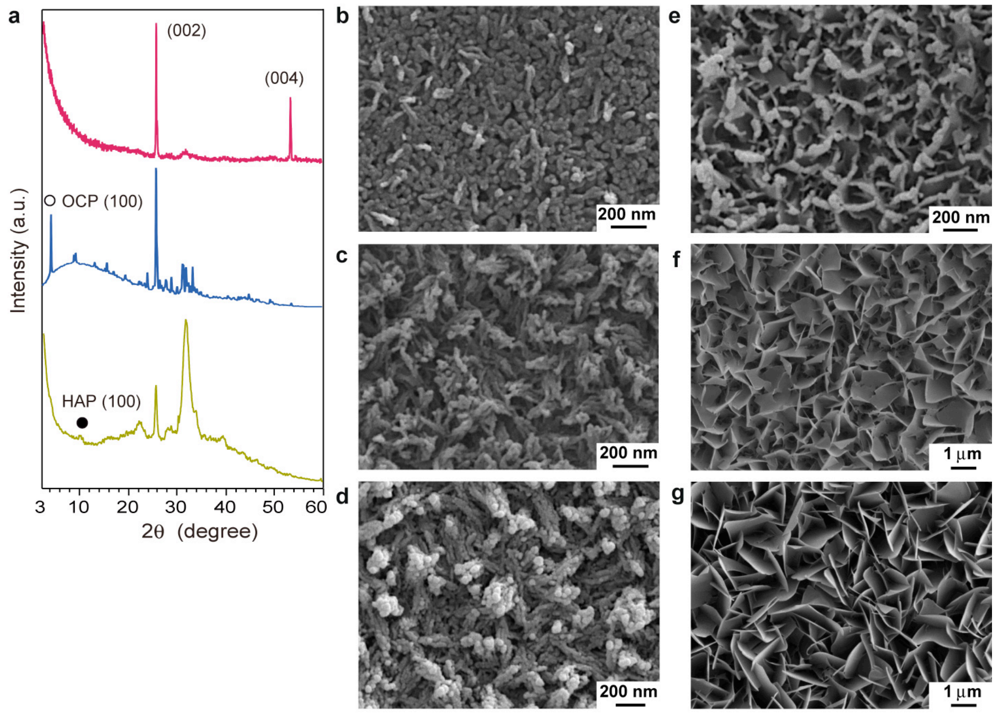

Supplementary Fig. S8. Characterization and time-resolved SEM observation of ACP substrate immersed in fluoride-less mother solution. (**a**) XRD (magenta) and micro-beam XRD (blue) patterns of grown layer and micro-beam XRD pattern of substrate (ochre) after 20-h immersion. Open circle indicates characteristic peak of OCP, and solid one indicates that of HAP. Top-view SEM images after (**b**) 1-min, (**c**) 3-min, (**d**) 5-min, (**e**) 10-min, (**f**) 30-min, and (**g**) 1-h immersion. Fragments precipitated in surface at 1 min increased in number and coalesced, forming root of tree structure between 3 and 5 min. Roots did not change to nanoparticles but to irregularly shaped plates at 10 min. Plates grew and showed the same morphology as those of final OCP between 30 min and 1 h.

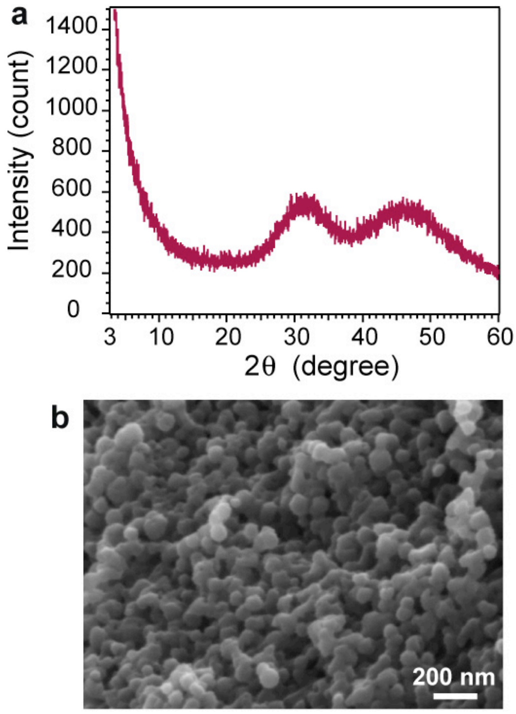

Supplementary Fig. S9. Characterization of ACC. (**a**) XRD pattern of ACC nanoparticles.

(**b**) Cross-sectional SEM image of compression-molded substrate.

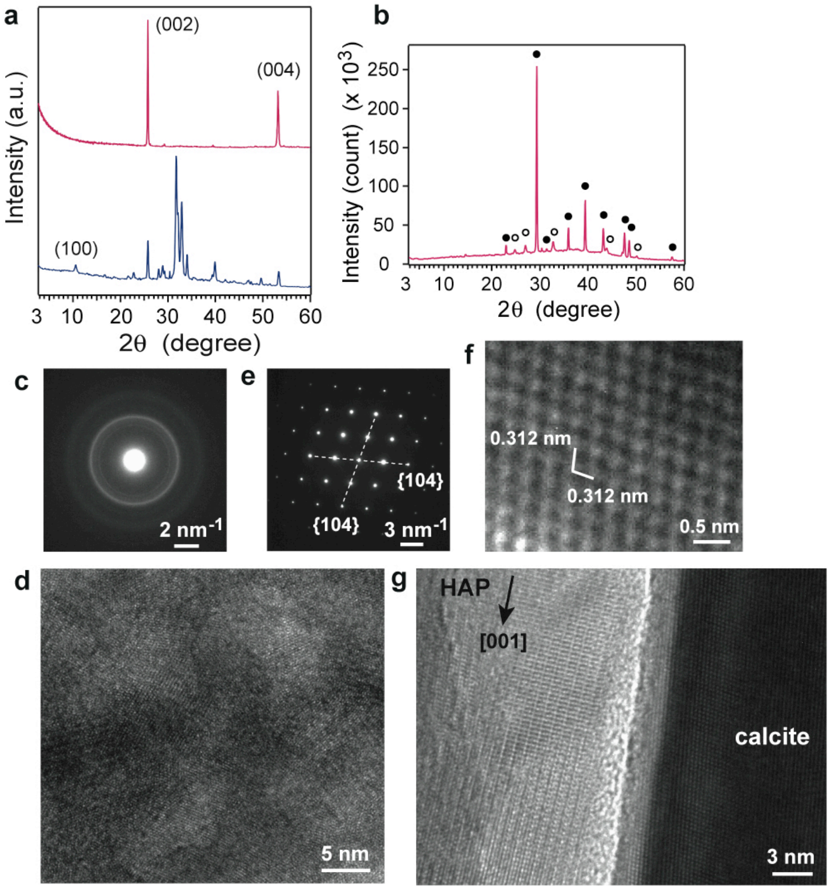

Supplementary Fig. S10. Characterization of ACC substrate immersed in mother solution for 20 h. (**a**) XRD (magenta) and micro-beam XRD (blue) patterns of GL (same patterns as those in Fig. 1e). (**b**) Micro-beam XRD pattern of substrate. Solid circles: calcite, open circles: vaterite. (**c**) SAED pattern of IL (FIB-prepared sample), consistent with that in Fig. 1l. (**d**) TEM image of HAP nanoparticles in intermediate layer. (**e**) SAED pattern of cubic crystal in substrate. Interplanar distances corresponding to calcite {104} were evident (viewed from <4–4–1>). (**f**) HR-TEM image corresponding to (**e**) (**g**) HR-TEM image of calcite with rod crystal on it. Lattice image of rod corresponds to that of HAP.

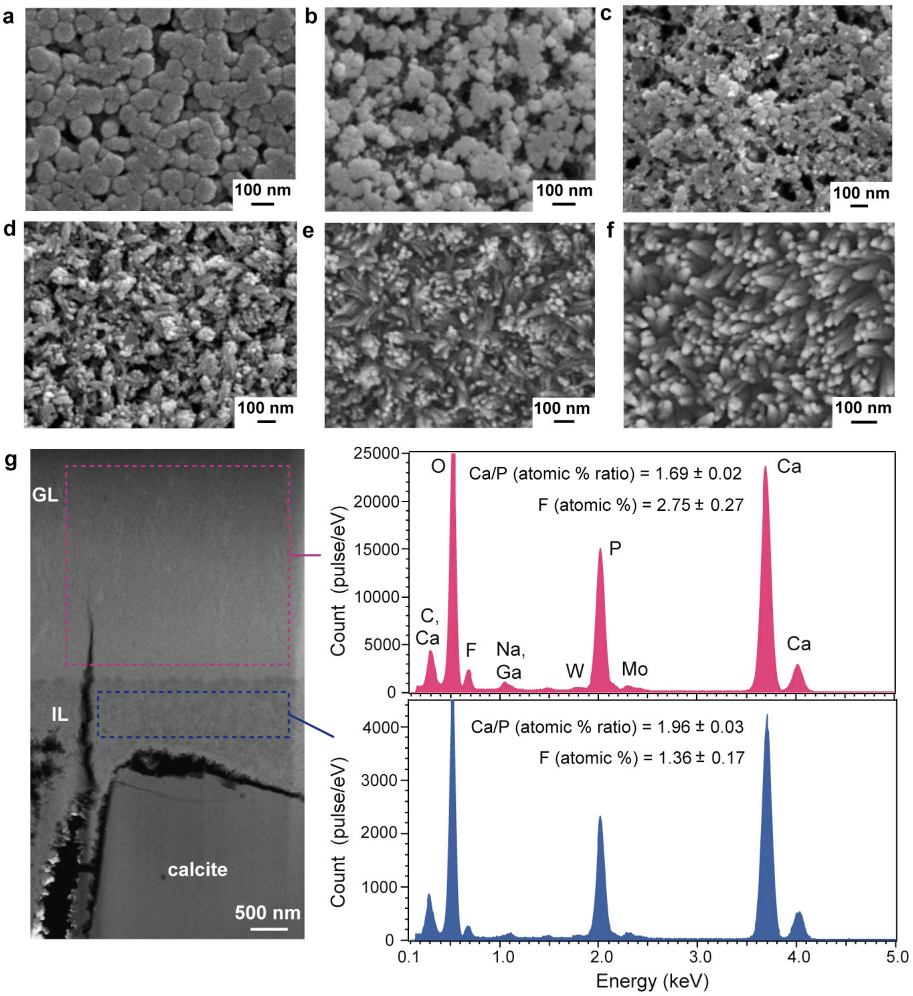

Supplementary Fig. S11. Time-resolved SEM observation and STEM-EDS analysis of ACC substrate immersed in mother solution. Time-resolved top-view SEM images observed in areas without calcite crystals at (**a**) 1 min, (**b**) 3 min (surface dissolution), (**c**) 5 min (surface dissolution), (**d**) 10 min (appearance of root of tree structure), (**e**) 30 min (nanoparticle elongation), and (**f**) 1 h (acute nanorod formation) after immersion. (**g**) Left: HAADF image of FIB-prepared sample using substrate immersed for 20 h. GL: grown layer, IL: intermediate layer. Dotted rectangle shows area analyzed. Right: Elemental analysis spectra.

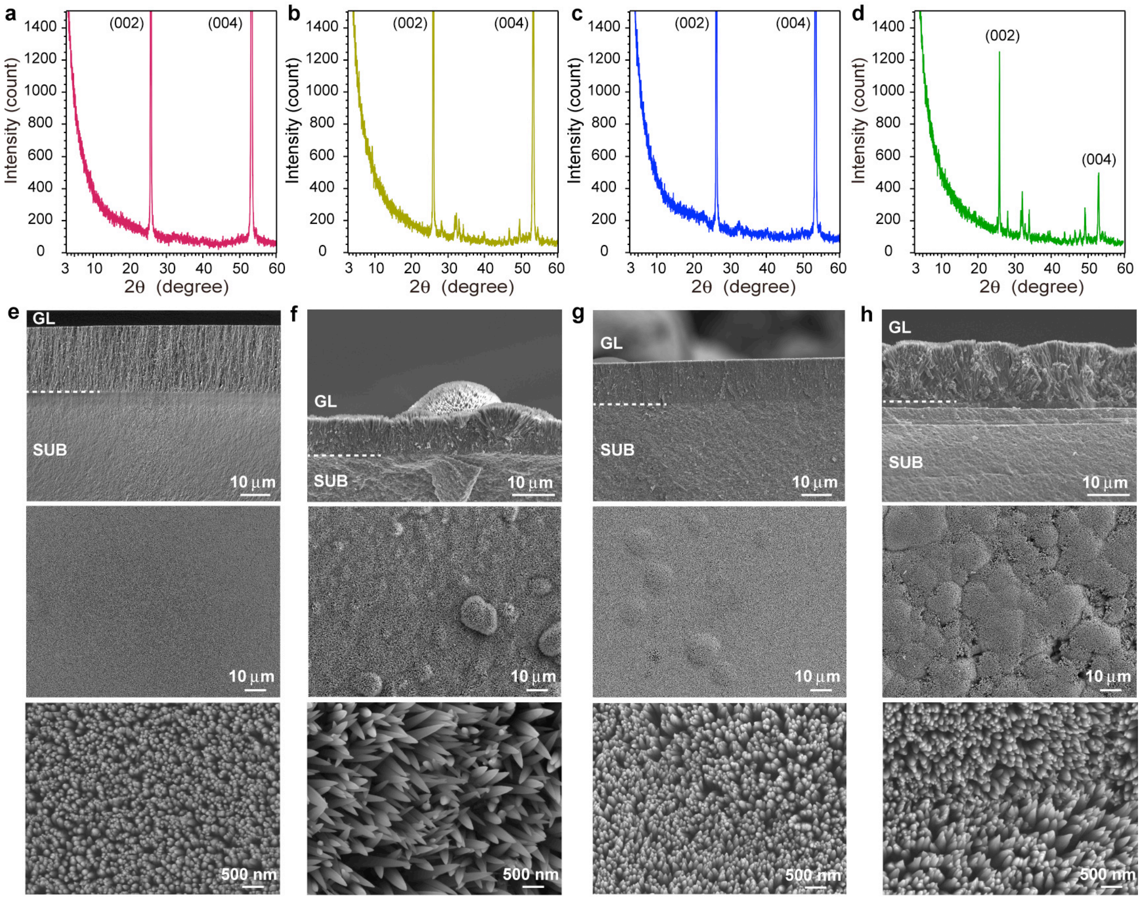

Supplementary Fig. S12. XRD measurement and SEM observation of ACP and crystalline nanoparticle's substrates immersed in mother solution for 20 h. XRD patterns of (**a**) ACP, (**b**) HAP, (**c**) β-tricalcium phosphate, and (**d**) TiO_2 substrates after immersion. Cross-sectional (upper), top-view low-magnification (middle), and top-view high-magnification (bottom) SEM images for (**e**) ACP, (**f**) HAP, (**g**) β-tricalcium phosphate, and (**h**) TiO_2 substrates after immersion (GL: grown layer, SUB: substrate). Spherulitic growth of nanorods was frequently observed except for ACP. Thickness and homogeneous growth of GL and tight packing and *c*-axis orientation degree of nanorods in GL for ACP substrate are excellent.
